# Simultaneous Detection of Common Founder Mutations Using a Cost-Effective Deep Sequencing Panel

**DOI:** 10.3390/genes15050646

**Published:** 2024-05-20

**Authors:** Sapir Shalom, Mor Hanany, Avital Eilat, Itay Chowers, Tamar Ben-Yosef, Samer Khateb, Eyal Banin, Dror Sharon

**Affiliations:** 1 Department of Ophthalmology, Hadassah Medical Center, Faculty of Medicine, The Hebrew University of Jerusalem, Jerusalem 91120, Israel; sapir.shalom@mail.huji.ac.il (S.S.); mor.hanany@mail.huji.ac.il (M.H.); aeilat@hadassah.org.il (A.E.); chowers@hadassah.org.il (I.C.); samerkhateb@gmail.com (S.K.); banine@cc.huji.ac.il (E.B.); 2 Department of Military Medicine and “Tzameret”, Faculty of Medicine, Hebrew University of Jerusalem and Medical Corps, Israel Defense Forces, Jerusalem 91120, Israel; 3 Rappaport Faculty of Medicine, Technion—Israel Institute of Technology, Haifa 3109601, Israel; benyosef@technion.ac.il

**Keywords:** NGS, panel sequencing, inherited retinal diseases, primers

## Abstract

Inherited retinal diseases (IRDs) are a clinically and genetically heterogeneous group of diseases which cause visual loss due to Mendelian mutations in over 250 genes, making genetic diagnosis challenging and time-consuming. Here, we developed a new tool, CDIP (Cost-effective Deep-sequencing IRD Panel) in which a simultaneous sequencing of common mutations is performed. CDIP is based on simultaneous amplification of 47 amplicons harboring common mutations followed by next-generation sequencing (NGS). Following five rounds of calibration of NGS-based steps, CDIP was used in 740 IRD samples. The analysis revealed 151 mutations in 131 index cases. In 54 (7%) of these cases, CDIP identified the genetic cause of disease (the remaining were single-heterozygous recessive mutations). These include a patient that was clinically diagnosed with retinoschisis and found to be homozygous for *NR2E3*-c.932G>A (p.R311Q), and a patient with RP who is hemizygous for an *RPGR* variant, c.292C>A (p.H98N), which was not included in the analysis but is located in proximity to one of these mutations. CDIP is a cost-effective deep sequencing panel for simultaneous detection of common founder mutations. This protocol can be implemented for additional populations as well as additional inherited diseases, and mainly in populations with strong founder effects.

## 1. Introduction

The Human Genome Project that was officially ended in 2004 [1] but successfully terminated on 31 March 2022 [2] was a breakthrough in human genetics as the entire human genome was sequenced for the first time. However, this project was long (over 12 years), costly (an estimated cost of USD 3 billion) and highly complicated to achieve (involved hundreds of researchers mainly from the USA and Europe) [3]. This emphasized the need for faster, higher-throughput, and cheaper methods. In subsequent years, many methods for parallel and deep sequencing, collectively termed next-generation sequencing (NGS), were developed by public companies such as pyrosequencing, Illumina, SOLiD and others. As a result, there is currently a large collection of possible kits and sequencing methods which enable one to perform comprehensive genetic analyses in modular approaches for various research and clinical purposes [1]. These developments currently enable the sequencing of a whole genome in a single day for less than USD 1000 [3], with the cost even being reduced to USD 100 per sample in recent announcements.

In addition to whole-genome sequencing (WGS), NGS is mainly being used as a tool for whole-exome sequencing (WES), panel sequencing, RNA sequencing, and more [4]. Each of these sequencing protocols has its advantage, and for each research purpose, a different protocol can be tailored.

Panel sequencing is based on targeting specific regions in the DNA, usually coding exons of a specific set of genes, and sequencing only those regions [5]. For example, a 25-gene panel for breast cancer enables the simultaneous sequencing of multiple cancer suspected genes, allowing for the identification of the genetic etiology in about 13.5% of cases [6]. Identifying the possible disease-causing mutations in individuals can help in prevention, early detection, and treatment. By using panel sequencing, a higher number of reads is obtained with a relatively low cost compared to WGS and WES due to the smaller regions that are sequenced [5]. However, such methods are relatively complex since libraries need to be designed, created and calibrated. This process includes library preparation of specific genomic locations and the design of special barcoded primers [7], a process that is unique for each panel and therefore each modification or update for such panels requires additional calibrations. As a result, most panels use available libraries and sequence a large number of genes resulting in general and non-specific panels. Moreover, the large number of genes in each panel leads to a massive amount of data, which makes for a more complicated sequencing and analysis process.

Although NGS is a complicated method, its efficiency and cost allow for a more comprehensive and accurate analysis compared to Sanger sequencing [8]. NGS allows one to obtain an overview of the studied genome and analyze the identified variants to better understand the cause of disease.

Inherited retinal diseases (IRDs) are a group of diseases which cause visual loss mainly due to photoreceptors’ degeneration or dysfunction [9]. As opposed to the majority of inherited diseases which are usually monogenic, each IRD is usually caused by pathogenic variants in multiple genes, and, currently, mutations in more than 250 genes are known to cause this group of diseases [10]. This leads to a great variety of genotypes, phenotypes and clinical appearances. For example, the most common IRD is retinitis pigmentosa (RP), which can be transmitted in all Mendelian modes of inheritance and can be caused by mutations in more than 90 genes. The great variety of mutations and genes in RP leads to a wide phenotypic variability. This makes the genetic diagnosis of these diseases very complicated, long, and inefficient [11]. We therefore present in the current study an NGS-based protocol for simultaneous screening of founder mutations, enabling us to detect 50 different founder mutations that cause IRDs in the Israeli and Palestinian populations.

## 2. Materials and Methods

### 2.1. Primer Design

**Figure 1:**
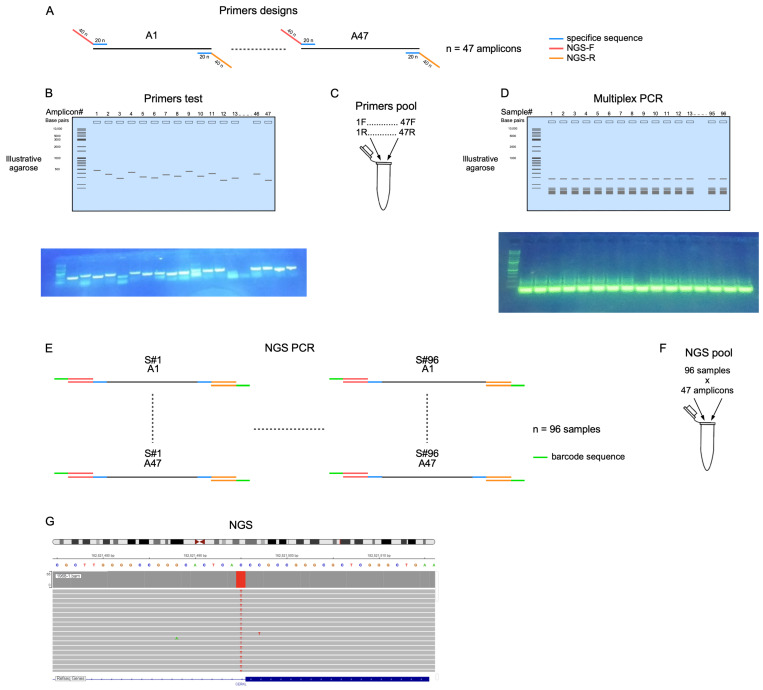
Scheme of CDIP strategy. (**A**). Primers designed for CDIP. Blue denotes the specificity region, and red and orange denotes the forward and reverse overhang adaptor sequences, respectively. (**B**). An agarose gel (an example is shown in the upper panel and an actual gel photo in the lower) of the initial primer test. Each well contains PCR amplification of a single set of primers. (**C**). Primer pool of all primer sets was used for multiplex PCR. (**D**). An agarose gel (an example is shown in the upper panel and an actual gel photo in the lower) of multiplex PCR products. Each lane represents PCR products of a single sample. A <100 bp smear that was generated from the large concentrations of primers can be seen. (**E**). Barcode sequences are added to each sample. (**F**). NGS pool is generated containing all the amplicons per patient. (**G**). The NGS results, showing an example from IGV for a homozygous variant.

The FastPCR software version 6.9.11 [12] was used to design primers for multiplex PCR using the following parameters: primer quality, temperature, primer length, primer amount, etc. The input FASTA file included sequences of regions harboring founder mutations with 300 bp upstream and downstream of each mutation. The mutation was highlighted and all the regions in which primers were allowed to be located were defined (Figure 1 and Supplement Figure S1). For the current study, 150 NGS cycles were used and therefore each founder mutation was localized up to 150 bp from the forward primer. To increase the chances for designing a suitable primer, a few possible locations for each primer pair and flexible parameters were allowed (including annealing temperature, primer design parameters, etc.).

An overhang adapter sequence was attached to the forward and reverse primers as previously described [13]. These adapter sequences were used to attach a specific barcode to each studied sample before sample pooling (Supplement Table S1). Prior to NGS analysis, each primer pair was tested by a standard PCR reaction, followed by agarose gel analysis to determine the specificity of the amplified PCR product (Figure 1).

### 2.2. Multiplex PCR and NGS

Primer pairs that were designed for multiple genomic regions were then combined to one test tube, followed by PCR, gel analysis and cleaning of PCR products by Exo-SAP. A second PCR amplification was then performed in order to add a unique barcode sequence (Nextera XT v2 forward PRIMER (N7) and Nextera XT v2 reverse PRIMER S5) to each of the 96 studied samples per run. The amplified products were pooled together and cleaned using Agencourt AMPure XP system (X1) and eluted in 50 μL elution buffer. Library purity and quantity were evaluated by 4200 TapeStation System using D1000 ScreenTape kit and by Qubit^®^ Fluorometer using Qubit dsDNA high-sensitivity assay, and the samples were sequenced using the NextSeq 500/550 machine (Illumina, San Diego, CA, USA) using the 150-cycle Mid Output kit. Since heterozygous variants might have as low as 25% of reads covering the mutant allele, we set the desired average coverage to 1000 reads, resulting in a minimum coverage of 100 reads. Therefore, the lowest possible heterozygous variant (with a coverage of only 100 reads and 25% represent the mutant allele) would be covered by 25 reads and the average heterozygous variant by 500 reads. This ensures the identification of all possible heterozygous variants. The actual average coverage (after filtering out low quality reads) was 914 reads per base.

### 2.3. Files Conversions

The NGS fastq output file was uploaded to Galaxy at https://usegalaxy.org/ (accessed on 18 May 2024), converted using “FASTQ Groomer”, and the sequences were aligned to the human genome by creating bam/bai files (using “Map with BWA-MEM”), which were then converted into two separate file formats: a coverage file (using “BedCov”) containing the number of reads for each target sequence; and a vcf file (using “FreeBayes”), which annotates the variants. The vcf files were uploaded to Franklin at https://franklin.genoox.com/ (accessed on 18 May 2024). Variant analysis was filtered based on the following criteria: frequency of the variant in the general population, location of the variant in the coding sequence, and quality (based on the number of reads). All pathogenic variants identified by the panel analysis were verified by Sanger sequencing of the PCR products.

## 3. Results

### 3.1. Selecting Founder Mutations for Analysis

**Figure 2:**
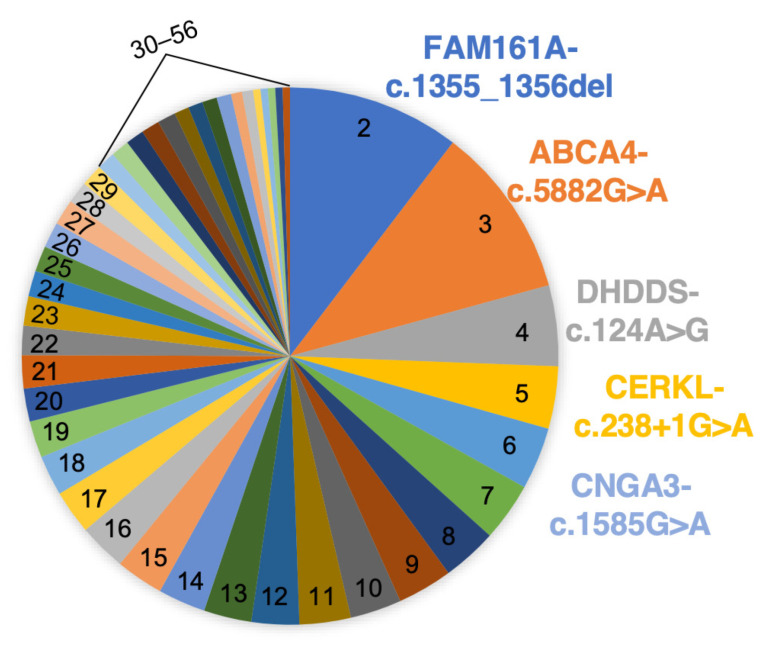
A pie chart showing the frequency of the 55 variants in our panel. The five most prevalent variants are mentioned. For each variant, the row number in Supplement Table S2 is shown.

As a first step towards generating a founder mutation panel, we tabulated founder mutations identified in our cohort of over 2000 families with IRDs and identified the most common ones (Figure 2, Supplement Table S2), a total of 49 mutations in 25 genes. Structural variants were excluded from this analysis. We then extracted the sequences flanking these mutations and generated a FASTA file for multiplex primer design. Multiplex primers were successfully designed using the FastPCR software version 6.9.11 for 47 out of the 49 target regions, while for two regions, no possible primers were suggested by the software. It should be noted that the screened regions included not only the 47 target founder mutations, but also other, less common mutations in the same amplicons, making a total of 61 mutations previously identified in the studied cohort.

### 3.2. Calibration of the NGS Panel

**Figure 3:**
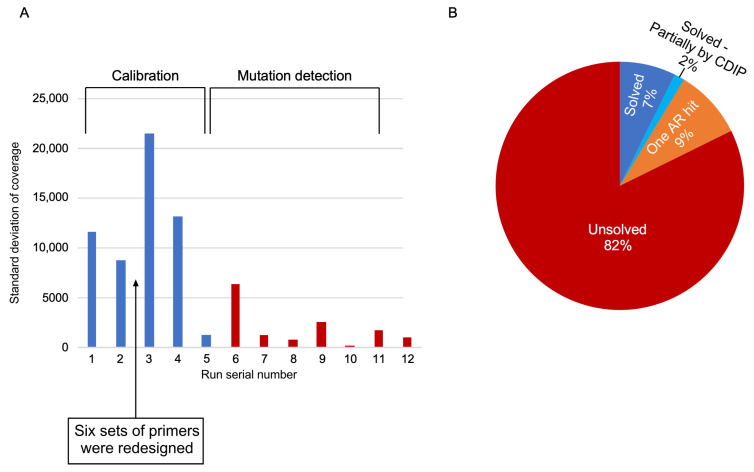
CDIP scaling and characterization. (**A**). A bar graph of the standard deviation of average coverage (*y*-axis) per each run (*x*-axis). Blue bars represent runs used for calibration and red bars represent runs used for mutation detection. (**B**). A pie chart showing the four genotyping categories: solved by CDIP, partially solved (one variant found using CDIP and the other using different methods), one AR variant, and no variants were found.

Since this protocol involves parallel sequencing, one can use the coverage results to calibrate the NGS procedure aiming to produce a more even distribution of reads across the 47 studied fragments. We therefore performed a series of calibration runs and downscaled or upscaled high- or low-represented amplicons, respectively. An initial test run of 96 samples revealed an over-representation of two overlapping *CNGA3* amplicons harboring neighboring mutations. To overcome this complication, we removed one of these overlapping fragments from the downstream analyses. We subsequently performed a calibration set of analyses by performing five runs on 4–48 samples each (Figure 3A). For each run, we calculated the average coverage of each amplicon. Amplicons with extremely poor or rich coverage were calibrated up or down in the following calibration run. The quality of each run was measured by the variance in coverage (standard deviation of coverage) among different amplicons (blue bars in Figure 3A). The small difference in the variance between the first two runs stems from minor corrections in primer concentration as part of the calibration process. However, six sets of primers did not yield any reads in the first two runs (although their primer concentration was elevated in the second run) and we therefore designed new primers for these six amplicons. These primers were included in run #3, causing a two-times increase in standard deviation. This was followed by two additional rounds of calibrations in runs #4 and #5, resulting in a drastic drop in variance to a level that is ~9% of the average variance in runs #1 through #4. This drastic reduction in variance indicated that the panel used in run #5 shows evenly distributed reads and can therefore be scaled up for 96-well experiments. We therefore performed a series of seven runs on 48 (run #5) or 96 samples (runs #6–#12) each.

### 3.3. Results of the IRD Panel of Founder Mutations

The panel includes 47 amplicons covering a total of 7050 bp in IRD genes, harboring 60 known mutations, mostly founder mutations in the Israeli and Palestinian populations, expecting to yield positive results in 31% (667/2147) of our current cohort of families with IRDs. We used this panel to screen a total of 787 IRD patients, and 740 (94%) of the samples yielded high quality results. Using the panel, we identified a total of 151 mutations in 131 (18%) index cases. One of these mutations is a hypomorphic allele (*ABCA4*-p.R943Q) that appeared in 23 cases but it appeared in trans with a pathogenic *ABCA4* mutation and was relevant for the disease in only one case. The genetic cause of 54 (7%) index cases was identified by CDIP and, in 10 cases, a heterozygous recessive mutation was identified by CDIP, while the second in-trans mutation was identified using other tools (Figure 3B). In 67 cases, a heterozygous AR pathogenic mutation was identified with no identified in-trans mutation.

### 3.4. CERKL Polymorphism

**Figure 4:**
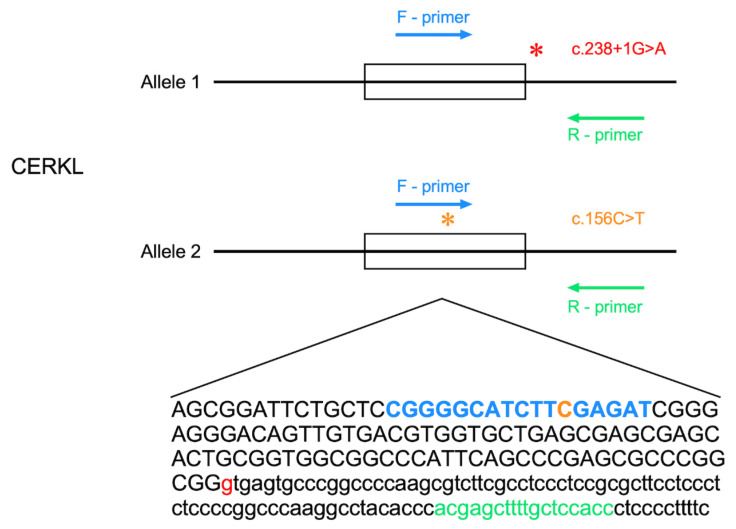
A polymorphism within the primer sequence affecting NGS results in the *CERKL* gene. In blue and green, the forward and revers primers, respectively. In red text and asterisk, the c.238+1G>A *CERKL* mutation. In orange text and asterisk, the c.156C>T polymorphism is located within the forward primer sequence.

Interestingly, four cases were genotyped as homozygous for the c.238+1G>T *CERKL* mutation, while Sanger sequencing clearly showed a heterozygous state. We performed whole-exome sequencing analysis on one of these cases (MOL1815-1) and indeed verified that the mutation is heterozygous. WES data analysis revealed that a heterozygous polymorphism (c.156C>T, p.F52F) is situated at position 14 out of the 20 nucleotide of the forward primer (Figure 4) and is in trans with the c.238+1G>T mutation. We therefore predict that the four cases with contradicting results share a relatively rare phenomenon in which a polymorphism in the primer binding site dramatically reduces binding affinity and therefore the number of WT reads was extremely low or even absent.

### 3.5. Interesting Patients

In a few cases, we identified disease-causing mutations that were not in line with the clinical diagnosis and needed to be further studied.

**Figure 5:**
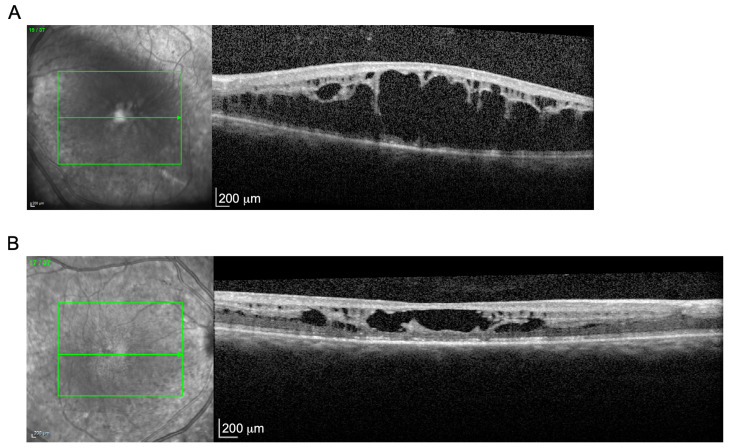
Optical coherent tomography (OCT) of the left eye (**A**) and the right eye (**B**) of a patient diagnosed with retinoschisis and found to be homozygous for an *NR2E3* mutation. The green arrow indicates the location of the OCT section.

MOL1118-1 is an isolate case who was clinically diagnosed at the age of 27 years with retinoschisis. She complained of nyctalopia starting at the age of 24. Her visual acuity was 0.10 decimal in both eyes (BE), anterior segments within normal limits, clear lens and in fundoscopy, macular schisis in BE was noticed. Full-field ERG analysis revealed a negative pattern in which the a-wave amplitudes (reflecting photoreceptor function) were within the normal limits, while the b-wave amplitudes (reflecting inner retinal function) were reduced. The cone flicker (30Hz) amplitudes were moderately reduced. Horizontal cross-sections of optical coherence tomography (OCT) showed typical retinoschisis, more severe in the left eye (Figure 5). Mutations in the X-linked gene, *RS1*, are the most common cause of retinoschisis, usually in male patients. CDIP revealed a previously published homozygous mutations, c.932G>A, p.R311Q in the *NR2E3* gene. This mutation has been reported as a relatively frequent cause of various IRDs, including RP, enhanced S-cone syndrome (ESCS), Goldmann–Favre syndrome, and clumped pigmentary retinal degeneration (CPRD). In a few cases, *NR2E3* mutations were also described in patients with retinoschisis [14,15,16].

MOL1964-1 is an isolate male of a mixed Ethiopian and Ashkenazi Jewish origin, who was diagnosed with RP at the age of 7 years with difficulties in night vision and constricted visual fields. His visual acuity at the age of 20 years was 0.5 in each eye. Funduscopic analysis revealed typical signs of RP including narrowed blood vessels, bone-spicule-like pigmentation, and pallor of the optic disc. CDIP was negative to all 60 studied mutations; however, a hemizygous missense variant, c.292C>A (p.H98N), was identified in *RPGR* exon 4. This variant was reported thus far in one case [17]. Another variant in the same nucleotide, c.292C>G (p.H98D), was previously reported to cause RP and to affect RPGR interaction with RAB28 and RPGRIP1. Another variant in the same codon, c.294C>A (p.H98Q), was previously reported to cause RP. This variant was inherited from the patient’s mother, who is of Ethiopian Jewish origin [18].

## 4. Discussion

Here, we present a cost-effective deep sequencing IRD panel (CDIP) for simultaneous detection of common founder mutations. The CDIP panel scheme can be used to build any panel of mutations that are relatively common as a cause of a genetic disease in a studied population. By using NGS, the cost of multiplex analysis of about 50 mutations per patient is as low as USD 10. Such panels can be used as a first screening tool for identifying the cause of disease, and only negative samples can be subsequently studied by more comprehensive methods, including gene panels and WES [19,20].

Based on the frequency of mutations in the Israeli and Palestinian populations studied here, the 61 mutations (including 47 founder mutations and 14 minor neighboring mutations) included in the panel are found in 31% of IRD patients and are responsible for the disease (sometimes in trans with a second mutation that is not included in the panel). In addition, it is possible to identify other mutations by chance, as we reported for the *RPGR* c.292C>A (p.H98N), which lies in proximity to one of the studied founder mutations. Our analysis revealed a lower detection rate of ~9% (64 out of 740) since many of the samples used for CDIP were prescreened for pathogenic mutations by Sanger sequencing and were therefore excluded from the current analysis if the causing mutation/s was/were identified.

One of the major contributions of CDIP would be to apply it to other populations and genetic diseases. To examine whether such IRD panels can be useful in other populations, we collected information reported on large cohorts of IRD patients [21]. The data show that the 50 most common mutations in each population are identified in 19–64% of IRD patients in the respective population (64% in Spain, 34% in North America, 24% in China, and 19% in England compared to 31% in the current cohort). Therefore, similar panels are likely to be useful in many other populations, especially those with strong founder effects.

## 5. Conclusions

To conclude, designing and generating NGS-based panels for the screening of common disease-causing mutations is a relatively easy and cost-effective task that can serve as the first mutation detection tool prior to more general and comprehensive tools, such as commercial panels and WES.

## Data Availability

Data of this study are presented within the article and its Supplementary Materials. The authors are willing to share materials, data sets, and protocols used in the acquisition of data presented in this publication with other researchers upon request (contact Dror Sharon, E-mail: dror.sharon1@mail.huji.ac.il).

## Supplementary Materials

The following supporting information can be downloaded at: https://www.mdpi.com/article/10.3390/genes15050646/s1, Figure S1: An example of a sequence that was used as an impot in FastPCR; Table S1: A list of CDIP primers; Table S2: A list of known variants covered by CDIP.
