# Supplementary material for: Simultaneous Detection of Common Founder Mutations Using a Cost-Effective Deep Sequencing Panel
Source: Genes (Basel). 2024 May 20;15(5):646. doi: 10.3390/genes15050646 (PMC11120920; doi:10.3390/genes15050646)
Supplement: Supplementary file 1 [file genes-15-00646-s001.zip › genes-2989680-supplementary/genes-2989680-supplementary.pdf]

SUPPLUMENTAL MATERIAL

Simultaneous Detection of Common Founder Mutations using a Cost-Effective Deep Sequencing Panel

Sapir Shalom[1,2], Mor Hanany[1], Avital Eilat[1], Itay Chowers[1], Tamar Ben-Yosef[3], Samer Khateb[1], Eyal Banin[1], Dror Sharon[1]

1- Department of Ophthalmology, Hadassah Medical Center, Faculty of Medicine, The Hebrew University of Jerusalem, 91120 Jerusalem, Israel.

2- Department of Military Medicine and "Tzameret", Faculty of Medicine, Hebrew University of Jerusalem and Medical Corps, Israel Defense Forces, Jerusalem, Israel.

3- Rappaport Faculty of Medicine, Technion - Israel Institute of Technology, Haifa, Israel.

Corresponding author:

Dror Sharon, PhD., Department of Ophthalmology, Hadassah-Hebrew University Medical Center, Jerusalem, Israel, 91120; Phone: +972 2 6777112; Fax: +972 2 6448917; E-mail: dror.sharon1@mail.huji.ac.il

Keywords: NGS, panel sequencing, Inherited retinal diseases, primers

Supp Figure S1

```
2. ABCA4- c.5882G>A, chr1:94486960C>G
TGTTCCCTGCTATTCATCTCGGGCACTGACACAGGGCCTCAGTGAGAATCACTCCAGCTG
AGCATCATTCCCTTTTCTGTGTTCTGTTTCTGCAGAGCATGGGTCAGCCTCGAGATGTCT
CAGTACTCACCACACCTCTGTGCCTGCCCATGTCAATATGTAACCTCCTAGTGCTGGTAG
TTTTC[TCCTAAACCATCCTTTGCTCTTTGTTCCCTCTTCCCCTCCTTGCTCTCACCCTGT
CTCAGTTCTCAGTCCGGTTTCTTCGTATCT]TGCAGATTTATCCAGGCACCTCCAGCCCAG
CAGTGGACAGGCTGTGTGTCGGAGTTCGCCCTGGAGAGGTGGGTACTCTG[CAGACCACGT
GTGAAAGGCTTCCGAACATCAGCTCTTGTGCCTGCCTCTCCTCCCCATAAGGCAGAGCTA]
TTCAATAGGAACATAATGCCATAATGCAAGTCACATATGTAATTTTAAATCTTCCACTAG
CCACATGAGAAAAGTAAAAAGAAAATAGGTAAAATTAATTTCATTAGTATTTTTTATTTT
```

An example of a sequence that was used as an input in FastPCR. In this figure one of the founder mutations from the panel is presented (marked in yellow). The brackets (marked in red) are used to define the preferred primer location. The software designs the primers in the areas bounded by the brackets. The selected primers are marked in green.
